# Surprising Anticancer Activities of Psychiatric Medications: Old Drugs Offer New Hope for Patients With Brain Cancer

**DOI:** 10.3389/fphar.2019.01262

**Published:** 2019-10-22

**Authors:** Chuanjun Zhuo, Zhiyuan Xun, Weihong Hou, Feng Ji, Xiaodong Lin, Hongjun Tian, Weifang Zheng, Min Chen, Chuanxin Liu, Wenqiang Wang, Ce Chen

**Affiliations:** ^1^Department of Psychiatry, School of Mental Health, Psychiatric Genetics Laboratory (PSYG-Lab), Jining Medical University, Jining, China; ^2^Department of Psychiatry, Wenzhou Seventh People’s Hospital, Wenzhou, China; ^3^Department of China-Canada Biological Psychiatry Lab, Xiamen Xianyue Hospital, Xiamen, China; ^4^Department of Psychiatric-Neuroimaging-Genetics and Morbidity Laboratory (PNGC-Lab), Nankai University Affiliated Anding Hospital, Tianjin Mental Health Center, Mental Health Teaching Hospital, Tianjin Medical University, Tianjin, China; ^5^Department of Biochemistry and Molecular Biology, Zhengzhou University, Zhengzhou, China; ^6^Department of Biology, University of North Carolina at Charlotte, Charlotte, NC, United States

**Keywords:** psychiatric drug, brain cancer, glioblastoma multiforme, psychiatric disorder, schizophrenia, bipolar disorder, depression

## Abstract

Despite decades of research and major efforts, malignant brain tumors remain among the deadliest of all cancers. Recently, an increasing number of psychiatric drugs has been proven to possess suppressing activities against brain tumors, and rapid progress has been made in understanding the potential mechanisms of action of these drugs. In particular, the traditional mood stabilizer valproic acid, the widely used antidepressants fluoxetine and escitalopram oxalate, and the atypical psychiatric drug aripiprazole have demonstrated promise for application in brain tumor treatment strategies through multiple lines of laboratory, preclinical, and clinical evidence. The unexpected discovery of the anticancer properties of these drugs has ignited interest in the repurposing of other psychiatric drugs to combat brain cancer. In this review, we synthesize recent progress in understanding the potential molecular mechanisms underlying the brain cancer–killing activities of representative psychiatric drugs. We also identify key limitations in the repurposing of these medications that must be overcome to enhance our ability to successfully prevent and treat brain cancer, especially in the most vulnerable groups of patients, such as children and adolescents, pregnant women, and those with unfavorable genetic variants. Moreover, we propose perspectives that may guide future research and provide long-awaited new hope to patients with brain cancer and their families.

## Introduction

Brain cancer is among the most lethal of all cancers. A large fraction of adult patients with glioblastoma multiforme (GBM), also known as glioblastoma, the most common and aggressive primary brain tumor, dies within 2 years of GBM diagnosis (Chinot et al., 2014; Gilbert et al., 2014). The treatment of brain cancer remains very challenging and is usually less clinically effective than desired. The blood-brain barrier (BBB) prevents many existing chemotherapeutic drugs from reaching the brain tissues, thereby restricting the treatment of brain tumors. Even systematically administered chemotherapeutic drugs that can freely penetrate the BBB are present at low concentrations in brain tumors, below levels required to achieve clinical effectiveness (Pitz et al., 2011; Gribkoff and Kaczmarek, 2017).

Over the past decades, psychiatric medications have been shown to be clinically effective in the treatment of a broad range of mental illnesses, and they have been targets for drug repurposing to increase cost effectiveness. Existing psychiatric drugs have several advantages, such as free penetration of the BBB and thorough study in clinical settings (Lee et al., 2016). Besides their antipsychotic activities, an increasing number of these medications has been proven to exhibit surprising anticancer effects on a range of malignant tumors, including those in the brain [e.g., valproic acid (VPA), fluoxetine (FLX), escitalopram oxalate, and aripiprazole (ARP)]. These new findings regarding the repurposing potential of psychiatric drugs have ignited interest in further exploration of the effects of other such drugs relevant to the treatment of brain cancer.

Rapid progress has also been made recently in the understanding of mechanisms potentially underlying the brain cancer–killing activity of certain psychiatric drugs. However, limitations of the repurposing of these drugs must be overcome to enhance our ability to successfully prevent and treat brain cancer, especially in the most vulnerable groups of patients, such as children, adolescents, pregnant women, and those with unfavorable genetic variants. In the present review, we summarize recent progress in understanding of the brain cancer–killing activity of representative psychiatric drugs and potential underlying mechanisms. Additionally, we identify potential obstacles that must be overcome to enhance our ability to successfully prevent and treat brain cancer and propose perspectives for future research.

## Psychiatric Drug-Use Influences Cancer Risk

Several epidemiological studies have shown that the incidence of cancer is significantly lower in patients with schizophrenia than in the general population (Grinshpoon et al., 2005; Chou et al., 2011). Additionally, several analyses have revealed decreased risks of the development of certain forms of cancer in patients with schizophrenia compared with control individuals (Cohen et al., 2002; Barak et al., 2005). Based on these findings, researchers have hypothesized that patients with schizophrenia may be protected against some cancer types (Cohen et al., 2002; Barak et al., 2005;<br/>Grinshpoon et al., 2005; Chou et al., 2011). Some have attributed this protection potentially to the use of antipsychotic drugs with antineoplastic effects, including phenothiazines, VPA, selective serotonin reuptake inhibitors (SSRIs), tricyclic antidepressants, and monoamine oxidase inhibitors, rather than to schizophrenia itself (Huang et al., 2018; Fond et al., 2012). The incidences of some forms of cancer (e.g., prostate and colorectal cancers) were found to be significantly lower in patients with schizophrenia treated with specific antipsychotic drugs than in control individuals (Mortensen, 1989; Mortensen, 1992; Lichtermann et al., 2001; Huang et al., 2018). Significant associations have been found between the use of specific antipsychotic drugs and a low risk of brain tumors, including GBM (Wang et al., 2017; Grinshpoon et al., 2005). Epidermal growth factor receptor (ErbB) and epidermal growth factor (EGF) as the ligand of ErbB have been found to be involved in the development of both gliomas and schizophrenia (Berezowska and Schlegel, 2011; Sotoyama et al., 2011; Iwakura and Nawa, 2013; Brocard et al., 2015); thus, psychiatric drugs targeting the EGF/ErbB signaling pathway can also be expected to affect gliomas in patients with schizophrenia. Most epidemiological studies conducted on this topic to date have focused on adults. Primitive neuroectodermal tumors of the central nervous system, including medulloblastomas, occur commonly in the cerebellums of children. Cancer risk in pediatric patients on psychiatric drugs has rarely been evaluated.

Cheng and colleagues (2015) found that thioridazine, a first-generation antipsychotic drug, had potent anti-GBM activities, mainly through the induction of endoplasmic reticulum (ER) stress and GBM cell autophagy. Wang et al. (2017) recently showed that quetiapine, an atypical antipsychotic drug, suppressed the proliferation of brain tumor cells and induction of the differentiation of glioblastoma-derived stem-like cells (GSCs) through inhibition of the Wnt/β-catenin signaling pathway. Clozapine, an atypical antipsychotic drug, has also been reported to suppress the proliferation of human GBM U87MG cells (Shin et al., 2006). Further studies showed that treatment with clozapine led to cell cycle arrest at the G0/G1 phase with reduction in cyclin D1 expression, which was mediated by the inhibition of protein kinase B (Akt) activation (Shin et al., 2006). ARP, an atypical antipsychotic drug used widely for the treatment of schizophrenia and schizoaffective disorder, has been proven to inhibit migration and induce apoptosis of glioma cells directly through the inhibition of Src kinase, a proto-oncogenic kinase protein (Kim et al., 2018a). All of these findings suggest that psychiatric drugs possess antitumor effects.

## Potential Molecular Mechanisms Underlying the Anticancer Properties of Specific Psychiatric Drugs in the Brain

Research teams throughout the world have extensively explored the molecular mechanisms underlying the anticancer activities of certain psychiatric medications, which may help to explain the reduced cancer incidence in patients taking these drugs and guide their repurposing for use against GBM and other forms of brain cancer. The characteristics of the representative psychiatric drugs with anticancer activity and potential mechanisms of action in the brain that are considered in this review are summarized in **Table 1**.

**Table 1:** Summary of representative psychiatric drugs with anticancer activity and potential mechanisms of action in the brain.

Psychiatric drugs	Primary use for adults, children or both	Type of brain tumors	Potential mechanism of anti-cancer activity
Antipsychotics Aripiprazole Clozapine Fluphenazine Olanzapine Quetiapine Thioridazine	Adults and children Schizophrenia, or bipolar disorder Adults but not older adults with dementia Schizophrenia Adults and children over 16 Schizophrenia Adults and children aged 13 and older Schizophrenia, or bipolar disorder Adults Schizophrenia, or bipolar disorder Adults with schizophrenia, or psychosis	Glioma Glioblastoma Glioma Glioblastoma Glioblastoma Glioblastoma	Inhibition of the migration and induction of apoptosis of glioma cells via Src oncogenic tyrosine kinase signaling Inhibition of cell proliferation; Induction of the autophagic cell death Induction of autophagic cell death via inhibition of phospahtidylinositol-3 kinase (PI3K)/protein kinase B (AKT)/mammalian target of rapamycin (mTOR) pathway Inhibition of proliferation via promoting activation of AMP-activated protein kinase (AMPK) Induction of differentiation of glioblastoma-derived stem-like cell via inhibition of the Wnt/nt hibiti signaling pathway Induction of endoplasmic reticulum (ER) stress and GBM cell autophagy
Mood stabilizers Valproic acid	Adults and children Bipolar disorder, epilepsy, or migraine headaches	Glioblastoma	Inhibition of cell proliferation by promoting cell cycle arrest; Induction of apoptosis via epigenetic modification of histone deacetylase; Reduction of invasiveness via Wnt/β catenin signaling pathway
Antidepressants Fluoxetine	Adults and children;	Glioblastoma	Promotion of apoptosis by binding to α-amino-3-hydroxy-5-methyl-4-isoxazolepropionic acid receptor (AMPAR) and inducing Ca^2+^ influx
Escitalopram oxalate	Adults and adolescents (12-17 years); Major depression disorder, anxiety disorders	Glioblastoma	Induction of autophagy and apoptosis; Inhibition of proliferation

### The Dopamine Receptor Pathway

The dopamine (DA) receptor pathway is a well-recognized neurotransmitter in the brain with critical roles in multiple important brain processes (e.g., cognition, motivation, and memory, pleasure) and behaviors in humans. Abnormal DA levels have been found in patients with psychiatric disorders and neurological diseases (Girault and Greengard, 2004). DA exerts its biological functions through DA receptors, which are classified into two families: D1-like receptors (D1 and D5 subtypes) and D2-like receptors (D2, D3, and D4 subtypes). The DA receptor pathway is required for both DA function and GBM proliferation. Li and colleagues (Li et al., 2014) found that the genes involved in GBM growth are also essential for DA receptor function. Using shRNA technology to interfere with the expression of target genes, the research team performed genome-wide screening for genes associated with GBM growth and found integrated molecular signaling between epidermal growth factor receptor and dopamine receptor D2 (DRD2) in GBM (Li et al., 2014). DA imbalance in the brain due to abnormal regulation of the DA receptor pathway has been considered to be a hallmark of many types of psychiatric disorder, such as attention deficit hyperactivity disorder and schizophrenia, and DA antagonists have been developed as a class of antipsychotic drugs to effectively treat patients with these disorders. Some DA antagonists have shown surprising anti-GBM effects in cell culture and mouse models (Li et al., 2014). Olanzapine, a second-generation antipsychotic drug, has been demonstrated to inhibit the proliferation of human GBM cells in a cell culture model.

### Epigenetic Modification of Histone Deacetylase

Epigenetic dysregulations, apart from genetic aberrations, are well-known drivers of the development and progression of malignant tumors, including GBMs (Lee et al., 2017). The mechanism by which psychiatric drugs exert anticancer effects in the brain may be epigenetic. Among epigenetic modifications (e.g., methylation, phosphorylation, acetylation, and ubiquitination), histone acetylation is the most common and important, and its involvement in carcinogenesis has been well established. Histone deacetylases (HDACs) and histone acetyltransferases are key enzymes in histone acetylation, and the inhibition of HDACs induces the differentiation and suppresses the proliferation of cancer cells through the modulation of early growth response 1; thus, HDAC inhibitors have been proposed as effective epigenetic therapy for cancer (Gottlicher et al., 2001; Sharma et al., 2010). In 2006, the United States Food and Drug Administration (FDA) approved vorinostat, a new anticancer drug that significantly inhibited abnormal chromatin remodeling in various cancer cells and was developed by targeting histone acetylation through HDACs.

Several studies have demonstrated the beneficial effects of VPA in patients with brain tumors (Blaheta and Cinatl, 2002; Chen et al., 2012; Osuka et al., 2012; Barker et al., 2013; Ruda et al., 2016). Retrospective clinical studies have shown that VPA prolongs overall survival in patients with GBM (Ruda et al., 2016). This FDA-approved psychiatric medication is currently being used as an HDAC inhibitor and has been shown to reduce the proliferation and to induce the apoptosis of brain cancer cells. Apoptosis is well understood to be involved in epigenetic regulation, such as the inhibition of histone acetylation. These findings suggest that treatment with the potent HAD inhibitor VPA may mediate cell death in brain tumor tissues (Osuka et al., 2012; Ruda et al., 2016; Huang et al., 2018).

### Voltage-Gated Ion Channels and Transporters

Researchers have noted that many psychiatric medications modulate neuronal excitability through common mechanisms involving voltage-gated ion channels and transporters. For example, valproate is well documented to potentiate GABA-ergic effects; inhibit voltage-gated sodium (Nav), potassium (Kv), and calcium (Cav) channels; and reduce N-methyl-D-aspartic–mediated responses, through which VPA modulates the excitation of neurons. The anti-GBM effects of VPA observed in several *in vitro* and *in vivo* studies support the results of clinical studies (Blaheta and Cinatl, 2002; Chen et al., 2012; Osuka et al., 2012), but the exact mechanisms underlying the antitumor effects of VPA remain unclear. Considering that deep reorganization of ion channel and transporter expression is observed in gliomas relative to astrocytes and other nonneoplastic cells in the brain and that these changes alter intracellular pH and [K+] control, glutamate uptake, and invasiveness, these primary mechanisms of action may lead to VPA-mediated anticancer effects, including the inhibition of cell proliferation by the promotion of cell cycle arrest, the inhibition of angiogenesis, and the induction of apoptosis (Osuka et al., 2012; Ruda et al., 2016; Huang et al., 2018). Clozapine, a second-generation antipsychotic drug, has been reported to induce the expression of chloride channel 4 (CLC-4), a member of the chloride channel/transporter family, in glioblastoma and neuroblastoma cells (Jeon et al., 2015). Following treatment with clozapine, CLC-4 expression was increased in a dose-dependent manner in neuroblastoma (SH-SY5Y) and glioma (U87) cells (Jeon et al., 2015). Moreover, the protein kinase A (PKA)/cAMP response element-binding protein (CREB) signaling pathway has been identified as a molecular mechanism by which clozapine stimulates CLC-4 (Jeon et al., 2015). CLC-4 has been found to be responsible for neurite outgrowth in PC12 cells (Hur et al., 2013). Thus, members of the chloride channel/transporter family members are critical for the modulation of apoptosis and proliferation of various cell types, and clozapine may suppress tumor growth and promote apoptotic events in glioma cells, partially through CLC-4 induction.

### Src Oncogenic Tyrosine Kinase Signaling

Src, also known as cellular-Src, is a proto-oncogene tyrosine kinase and an important regulator of intracellular signal-transduction pathways. Src pathway activation is tightly regulated under normal conditions; its perturbation or excessive activity has been observed frequently in patients with many forms of malignancy, and constant Src activation in malignant tissues has been shown to promote proliferation, invasion, metastasis, and angiogenesis (Tsatsanis and Spandidos, 2000; Fizazi, 2007; Wheeler et al., 2009). The inhibition of Src activity with antagonists has been used in the development of drugs for cancer treatment (Wheeler et al., 2009). ARP is an atypical antipsychotic drug used widely to treat schizophrenia and psychotic episodes in patients with bipolar disorders, depression, and other mental illnesses (Kane et al., 2002). Using cell culture and xenograft mouse models, Kim et al., (2018a) found that ARP significantly restricted the migratory capacity of U251 glioma cells, and the effect was associated with the regulation of matrix metalloproteinase-9 (MMP-9), a member of the zinc-metalloproteinase family involved in the degradation of extracellular matrix and pathological processes such as metastasis. They further determined that APR acts directly on Src, resulting in disturbance of the activation of Src oncogenic tyrosine (Kim et al., 2018a). Thus, Src modulates a signaling cascade or pathways (e.g., phosphorylated phosphatidylinositide 3-kinase, signal transducer and activator of transcription 3, and Akt) involved in glioma cell proliferation and migration (Kim et al., 2018a).

### The Wnt/β-Catenin Signaling Pathway

Studies of the role of the Wnt/β-catenin signaling pathway in the context of brain tumors have generated conflicting results. Several studies have shown that the overexpression of β-catenin reduces the proliferation and migration of glioma cells and the expression of stem cell markers, and others have shown that the enhancement of Wnt expression stimulates the self-renewal and proliferation of brain tumor cells (Kotliarova et al., 2008; Nowicki et al., 2008; Korur et al., 2009). Rampazzo et al. (Rampazzo et al., 2013) found that Wnt/β-catenin mediated the reprogramming of GBM cells toward a neuronal-like fate. Intriguingly, a recent study demonstrated that VPA can affect the canonical Wnt/β-catenin pathway (Riva et al., 2018), a highly conserved signaling pathway that has been thought to play pivotal roles in stem cell self-renewal and embryogenesis modulation (Nusse et al., 2008; van Amerongen and Nusse, 2009; Riva et al., 2018) and whose aberrant activation has been identified in a range of cancers (Duchartre et al., 2016; Zhan et al., 2017), including GBM (Zhang et al., 2012). Impairment of the canonical Wnt signaling pathway is observed frequently in patients with GBM (Zhang et al., 2012) and is associated closely with GBM development, invasiveness, and progression (Gong and Huang, 2012; Kahlert et al., 2012; Kierulf-Vieira et al., 2016).

### Autophagy

Autophagy, characterized by excessive self-degradation, is a highly efficient form of cell death. The induction of autophagy is a novel strategy in the development of anticancer therapies (Moretti et al., 2007; Guo and White, 2016; Chen et al., 2018). Escitalopram oxalate, a traditional SSRI, significantly inhibited GBM proliferation in cell cultures, which was associated with the induction of apoptosis cascades in these cells (Chen et al., 2018). Escitalopram oxalate also reduced the invasive capacity of U87MG cells in xenografted BALB/c nude mice (Chen et al., 2018). Chen and colleagues (2018) found that escitalopram oxalate induced autophagy in GBM8401 cells, indicating that the drug inhibits different brain tumor cell lines through diverse mechanisms. Further studies revealed cross talk among autophagy, ER stress–mediated apoptosis, and the NF-kappa B pathway in GBM cells, resulting in the regulation of the cells’ destiny and the sensing of changes in the tumor microenvironment. Most recently, Zappavigna et al. (2019) reported that EA0100C red, a hydroquinone-based derivative, elicited autophagy through activation of a ROS-dependent unfolded protein response in GBM, which may be the basis of its antitumor effects. A recent study showed that the antitumor activity of miR-24 was due, at least in part, to effects on glioma cell autophagy and viability (Chen et al., 2019). In addition to escitalopram oxalate, other psychiatric medications, including clozapine and fluphenazine, have been recently reported to induce autophagic cell death (Shen et al., 2016; Kim et al., 2018b). Fluphenazine activated autophagic cell death in U87 glioma cells by inhibiting the phospahtidylinositol-3 kinase/Akt/mammalian target of rapamycin signaling pathway (Shen et al., 2016; Kim et al., 2018b). Although the mechanisms underlying cross talk between autophagy and other cellular processes and signaling pathways remain to be further investigated, these findings have important implications for progress toward GBM treatment *via* the induction of autophagy. Notably, escitalopram oxalate’s induction of autophagy in GBM8401 cells in culture and animal models suggests the potential of repurposing this drug for the treatment of GBM (Chen et al., 2018).

## Perspective for the Repurposing of Psychiatric Drugs to Combat Brain Cancer: Opportunities and Challenges

With accumulating new discoveries regarding the potential application of psychiatric drugs in the treatment of brain cancer, several key challenges have been identified. These obstacles must be overcome to achieve more effective prevention and treatment of brain cancer.

First, little research has examined the repurposing of psychiatric medications for brain cancer treatment in children and pregnant women. Brain cancer is the most common form of solid tumor in children and the leading cause of pediatric cancer–related death (Sturm et al., 2017). GBM accounts for up to 20% of solid tumors in children, much higher than the approximately 4–5% of solid tumors in adults (Chinot et al., 2014; Gilbert et al., 2014; Sturm et al., 2017). Surgical resection remains the first-line treatment option for primary GBMs in children, but it can be challenging because some brain tumors are located in areas beyond the reach of neurosurgical intervention (Sturm et al., 2017). The exposure of children to radiation has a profound impact on brain development, with serious adverse effects that can extend to adulthood (Viswanathan et al., 2011; Sturm et al., 2017). In addition, fewer chemotherapeutic agents are available for pediatric than for adult patients with brain cancer, due in part to the difficulty of acquiring single-center data for the evaluation of drug safety and efficacy in the pediatric population. Furthermore, fewer psychiatric medications are available for pediatric than for adult patients (Krause et al., 2018). VPA is among the psychiatric drugs that have been proven in preclinical and clinical studies to be safe for children (Azorin and Findling, 2007); further examination of the broad spectrum of anticancer activities of these drugs in children is needed to enable the development of therapeutic approaches for pediatric brain tumors.

Second, scientific studies of the repurposing of psychiatric drugs for patients with GBM and unfavorable genetic variants are lacking. Variants in genes encoding the DA receptors may impact the anticancer effects of antipsychotic drugs. For example, *Taq* IA (rs1800497) is the most thoroughly studied single-nucleotide polymorphism associated with a wide range of psychotic disorders (Grandy et al., 1989; Lewis et al., 2003). It is located on chromosome 11q22–q23 of the *DRD2* gene and consists of a single nucleotide change (C/T), with the two alleles referred to as A2 (C) and A1 (T) (Grandy et al., 1989; Lewis et al., 2003). However, whether this and other variants (Grandy et al., 1989; Lewis et al., 2003; Bombin et al., 2008) alter the anticancer effects of psychiatric drugs and how these drugs can be appropriately repurposed for patients with GBM and such variants remain to be investigated.

## Conclusions

The surprising findings regarding the antitumor effects of psychiatric drugs in the brain are of clinical importance. In particular, these psychiatric drugs hold potential for repurposing for the treatment of patients with brain cancers, such as GBM. The successful treatment of GBM with current conventional therapies is very challenging for the following main reasons: GBM cells are highly resistant to these therapies; the brain tissues, especially those of children and adolescents, are highly susceptible to damage by these therapies; and the brain has very limited self-repair capacity. As the clinical characteristics of these psychiatric drugs, such as safety and toxicity, have been studied extensively, they are likely to bypass lengthy preclinical studies. Furthermore, unlike the majority (90%) of existing drugs, these psychiatric drugs can successfully penetrate the BBB. Thus, these psychiatric medications with anticancer activities offer new hope for patients with brain cancer. However, greater understanding of the mechanisms of action of their anticancer effects is needed. Multicenter studies of psychiatric drug repurposing for the treatment of primary and metastatic brain cancer in vulnerable groups, such as children, adolescents, and pregnant women, are needed.

## Author Contributions

CZ, ZX, and WH designed the idea; FJ, XL, and HT acquied the references; WZ, MC, CL, CC, and WW made a draft for this work; CZ, ZX, and WH performed the final version of this work.

## Funding

This work was supported by grants from the National Natural Science Foundation of China (81871052 to CZ), the Key Projects of Natural Science Foundation of Tianjin, China (17JCZDJC35700 to CZ), the Tianjin Health Bureau Foundation (2014KR02 to CZ), the Tianjin Anding Hospital Research Award 300000 yuan for CZ, and the Wenzhou Science and Technology Project (ZS2017011 to XL).

## Conflict of Interest

The authors declare that the research was conducted in the absence of any commercial or financial relationships that could be construed as a potential conflict of interest.
